# The origin, dissemination, and molecular networks of HIV-1 CRF65_cpx strain in Hainan Island, China

**DOI:** 10.1186/s12879-024-09101-w

**Published:** 2024-03-01

**Authors:** Dee Yu, Kaokao Zhu, Mu Li, Fei Zhang, Yuan Yang, Chunyun Lu, Shanmei Zhong, Cai Qin, Yanan Lan, Jipeng Yu, Jindong Ding Petersen, Junjun Jiang, Hao Liang, Li Ye, Bingyu Liang

**Affiliations:** 1 Guangxi Key Laboratory of AIDS Prevention and Treatment & Guangxi Colleges and Universities Key Laboratory of Prevention and Control of Highly Prevalent Diseases, School of Public Health, Guangxi Medical University, 22 Shuangyong Road, Nanning, 530021 China; 2 International School of Public Health and One Health, Hainan Medical University, 3 Xueyuan Road, Haikou, 571199 China; 3 Prevention and Treatment Department, the Fifth People’s Hospital of Hainan Province, 3 Xueyuan Road, Haikou, 570102 China; 4 Guangxi Engineering Center for Organoids and Organ-on-chips of Highly Pathogenic Microbial Infections & Biosafety laboratory, Life Science Institute, Guangxi Medical University, 22 Shuangyong Road, Nanning, 530021 China; 5 Guangxi medical university oncology school, 22 Shuangyong Road, Nanning, 530021 China; 6 The First Clinical Medical College, Guangxi Medical University, 22 Shuangyong Road, Nanning, 530021 China; 7 Research Unit for General Practice, Department of Public Health, University of Copenhagen, Copenhagen, Denmark; 8 Research Unit for General Practice, Department of Public Health, University of Southern Denmark, Odense, Denmark

**Keywords:** HIV-1, CRF65_cpx, Molecular network, Phylodynamic analysis, Hainan Island

## Abstract

**Background:**

HIV-1 CRF65_cpx strain carries drug-resistant mutations, which raises concerns about its potential for causing virologic failure. The CRF65_cpx ranks as the fourth most prevalent on Hainan Island, China. However, the origin and molecular epidemiology of CRF65_cpx strains in this area remain unclear. This study aims to estimate the spatial origins and dissemination patterns of HIV-1 CRF65_cpx in this specific region.

**Methods:**

Between 2018 and 2021, a total of 58 *pol* sequences of the CRF65_cpx were collected from HIV-positive patients on Hainan Island. The available CRF65_cpx *pol* sequences from public databases were compiled. The HIV-TRACE tool was used to construct transmission networks. The evolutionary history of the introduction and dissemination of HIV-1 CRF65_cpx on Hainan Island were analyzed using phylogenetic analysis and the Bayesian coalescent-based approach.

**Results:**

Among the 58 participants, 89.66% were men who have sex with men (MSM). The median age was 25 years, and 43.10% of the individuals had a college degree or above. The results indicated that 39 (67.24%) sequences were interconnected within a single transmission network. A consistent expansion was evident from 2019 to 2021, with an incremental annual addition of four sequences into the networks. Phylodynamic analyses showed that the CRF65_cpx on Hainan Island originated from Beijing (Bayes factor, BF = 17.4), with transmission among MSM on Hainan Island in 2013.2 (95%HPD: 2012.4, 2019.5), subsequently leading to an outbreak. Haikou was the local center of the CRF65_cpx epidemic. This strain propagated from Haikou to other locations, including Sanya (BF > 1000), Danzhou (BF = 299.3), Chengmai (BF = 27.0) and Tunchang (BF = 16.3). The analyses of the viral migration patterns between age subgroups and risk subgroups revealed that the viral migration directions were from "25–40 years old" to "17–24 years old" (BF = 14.6) and to "over 40 years old" (BF = 17.6), and from MSM to heterosexuals (BF > 1000) on Hainan Island.

**Conclusion:**

Our analyses elucidate the transmission dynamics of CRF65_cpx strain on Hainan Island. Haikou is identified as the potential hotspot for CRF65_cpx transmission, with middle-aged MSM identified as the key population. These findings suggest that targeted interventions in hotspots and key populations may be more effective in controlling the HIV epidemic.

**Supplementary Information:**

The online version contains supplementary material available at 10.1186/s12879-024-09101-w.

## Introduction

Human immunodeficiency virus type 1 (HIV-1) is classified into four distinctive phylogenetic groups, namely M (major), N (new), O (outlier), and P [1]. Of these, HIV-1 group M is particularly significant and includes a broad range of distinct subtypes (A-D, F–H, J, and K), along with circulating recombinant forms (CRFs) and unique recombinant forms (URFs), ultimately initiating the ongoing pandemic [2]. The uneven global distribution of various HIV-1 subtypes and CRFs can be attributed to varying founder effects, followed by localized dissemination driven by socioeconomic and behavioral factors [3], sometimes influenced by continuous influxes of new infections from neighboring regions [4]. The quest for a globally effective vaccine is in progress [5], but is challenged by the rapid genetic evolution and recombination of HIV, which are impacted by genetic, social, and epidemiological variables.

As of October 30, 2023, the Los Alamos HIV database has documented a total of 140 CRFs for HIV-1. In China, CRF01_AE, CRF07_BC, and CRF08_BC represent the predominant CRFs [6]. Most of the newly reported HIV patients (69.2%) on Hainan Island were needle-sharing drug users, with CRF01_AE (84.3%) as the dominant genetic form in 2009 [7]. In 2023, CRF01_AE (68.9%) also represented the main genetic form among patients with virologic failure in antiretroviral therapy (ART) on Hainan Island, followed by CRF07_BC. However, no comprehensive studies have been conducted in the past ten years regarding HIV-1 epidemic genetic forms in HIV-positive patients on Hainan Island [8].

CRF65_cpx emerged as a distinct CRF, first identified by Feng et al. in 2013 in western Yunnan Province, China. This CRF consists of genetic components from three genetic forms: B', C, and CRF01_AE [9]. A previous study demonstrated that CRF65_cpx likely originated around the year 2000 among heterosexuals (HETs) in Yunnan Province [10]. Over the subsequent years, transmission extended to men who have sex with men (MSM) in Beijing and Anhui, occurring approximately 3 to 7 years later. Subsequently, the CRF65_cpx strains propagated into other areas, including Hebei [11], Jiangsu, Heilongjiang, Jilin [12], and Guangxi [13]. In 2019, Ran Zhang et al. confirmed the presence of CRF65_cpx in approximately 0.8% of HIV-positive MSM across 19 cities located in six provinces of China [14]. Our previous study indicated that CRF65_cpx ranked fourth among patients with virologic failure in antiretroviral therapy on Hainan Island [8]. These findings underscore the national expansion of CRF65_cpx [15].

During its transmission, the CRF65_cpx strain has undergone changes in certain amino acid sites and cytotoxic T lymphocytes (CTL), potentially accelerating the progression of HIV-related diseases [16]. Genotypic resistance analyses revealed the presence of natural mutations, such as V179D and K103R/V179D, which are associated with CRF65_cpx resistance to nonnucleoside reverse transcriptase inhibitors (NNRTIs) [15]. While individually, V179D and K103R are relatively common polymorphic mutations with limited impact on NNRTIs susceptibility [17], their combination results in an approximately 10- to 15-fold reduction in susceptibility to efavirenz (EFV) and nevirapine (NVP) [18]. Previous studies have demonstrated a positive correlation between the rate of HIV-1 virus evolution and the advancement of the disease [19, 20]. Furthermore, a significant heterogeneity rate is visible both between and among various subtypes [21]. However, no previous reports are available on the evolutionary rate of CRF65_cpx.

Hainan Island, the southernmost province of China, has garnered popularity as a tourist destination due to its pleasant tropical climate. It has historically been regarded as an area in China with relatively low HIV prevalence. However, the dynamics of immigration and tourism have created various challenges in this area. Our study, encompassing the analysis of 58 HIV-1 CRF65_cpx polymerase (*pol*) sequences on Hainan Island, revealed that the CRF65_cpx strain played a significant role in driving the local HIV-1 prevalence. Here, we present a comprehensive account of the origin and dissemination pattern of the CRF65_cpx strain on Hainan Island using HIV-TRACE and Bayesian analyses. The findings can potentially inform the development of effective HIV surveillance strategies and public health interventions, specifically focusing on key populations. Furthermore, we offer valuable insights for enhancing HIV testing initiatives, monitoring drug resistance, and designing vaccines to control the spread of CRF65_cpx or other CRFs in the Hainan region and across China.

## Methods

### Study design and specimen preparation

An HIV molecular epidemiology and drug resistance monitoring study was conducted at the Fifth People's Hospital of Hainan Province from January 2018 to November 2022. The study recruited 1742 HIV-positive individuals who were either ART-naive or ART-experienced. In accordance with national standards, patient self-assessment interviews were used to gather demographic and epidemiological information. To adhere to ethical standards in China, the blood samples were linked to demographic and clinical data via a unique numerical code.

The viral RNA was extracted, and the HIV-1 *pol* gene was amplified at the Guangxi Key Laboratory of AIDS Prevention and Treatment (Guangxi Medical University, Guangxi, China). Subsequently, the HIV-1 *pol*, encompassing the entire protease (PR) and a segment of the reverse transcriptase (RT) (nucleotides 2253 to 3334 in reference strain HXB2; 1060 bp long), were sequenced by Sangon Biotech Company. Previously described primers were used to amplify the HIV *pol* gene using nested RT-PCR [22]. Finally, 1410 available *pol* sequences were obtained, including 58 (4.11%, 58/1410) CRF65_cpx sequences.

### Sequence alignment and subtype assignment

Sequencher v5.1.4.6 was used to assemble the HIV-1 *pol* sequences, while the MAFFT program was employed for alignment with the HXB2 reference sequence via the online HIV align tool (http://www.hiv.lanl.gov/content/sequence/viralign.html) [23]. The HIV-1 subtypes were determined using the online subtyping tool COMET (https://comet.lih.lu/) for preliminary classification [24], and identified by a phylogenetic maximum likelihood tree (ML tree). The general time-reversible (GTR) substitution with invariant sites (I) and gamma distributed (G) model in FastTree v2.2.10 was used to construct the ML tree (711 sequences) [25], which contains all available *pol* sequences of subtype C from China and of CRF65_cpx, as well as reference sequences of other genetic forms (A1, A2, B, B', D, K, F1, F2, H, N, CRF01_AE, CRF07_BC, CRF08_BC and other CRFs) from the Los Alamos HIV Sequence Database (http://www.hiv.lanl.gov/, accessed on October 30, 2023). Shimodaira-Hasegawa-like values were used to assess the reliability of the tree [26]. Sequences clustering with the reference sequences and bootstrap values ≥ 80% were identified as belonging to the same genetic form as the reference sequences. Group N (AJ006022, AJ271370, and AY532635) was set as the outgroup. The results were visualized using FigTree v1.4.3.9 (Table S1 and Fig. S1).

### Molecular transmission network analysis

To observe the network growth trend from 2018 to 2021, we constructed a molecular transmission network with a genetic distance threshold of 0.5% substitutions per site, corresponding to a maximum of around 2–3 years of viral evolution separating these strains [27]. Briefly, the *pol* sequences were aligned to the HXB2 sequence using online HIV align tool. The aligned sequences were then uploaded into HIV Transmission Cluster Engine (HIV-TRACE) to calculate the pairwise Tamura-Nei93 (TN93) genetic distance among all sequence pairs [28]. Finally, the network was visualized using Cytoscape v3.6.1. In the network, a node represents an individual. Nodes were linked to each other to construct a cluster (consisting of ≥ 2 sequences) if their pairwise genetic distance was below the threshold. To observe the growth of the clusters, the 2012–2018 sequences (year of diagnosis) were designated as baseline, after which the 2019, 2020 and 2021 sequences were introduced to construct the molecular networks.

### Sequence dataset and bayesian phylodynamic inference

Given the absence of breakpoints in CRF65_cpx *pol*, the possibility that some CRF65_cpx sequences were misclassified cannot be excluded. In a previous study [29], 15 CRF65_cpx sequences from the BLAST matches in the HIV Sequence Database (http://www.hiv.lanl.gov/content/sequence/HIV/mainpage.html) were misclassified as subtype C in 2019. Therefore, to identify as many CRF65_cpx *pol* sequences as possible from the HIV Sequence Database, all the subtype C and CRF65_cpx *pol* sequences (HXB2:2253–3334) from China and *pol* sequences of partly other subtypes (http://www.hiv.lanl.gov/, accessed on October 30, 2023), were downloaded. The downloaded sequences were selected based on the following inclusion criteria: (1) sequences already published in peer-reviewed journals, (2) no uncertainty about subtype assignment, (3) sampling time and city/province of origin were clearly established in the original publication, (4) the length of nucleotides ≥ 1000 bp, and (5) the proportion of nucleotides with ambiguity ≤ 0.5%. Finally, the global search yielded 83 available CRF65_cpx *pol* sequences, including 53 misclassified as subtype C (Table S1). The codons with drug resistance mutations (detected via the Stanford University HIV Drug Resistance HIVdb program at https://hivdb.stanford.edu) were manually removed from the alignment to avoid the confounding factor of convergent evolution in the phylogeny inference [30].

To infer the potential origins of the CRF65_cpx on Hainan Island, Bayesian Evolutionary Analysis by Sampling Trees (BEAST) was conducted using 141 available CRF65_cpx *pol* sequences (Dataset-1), which included 83 sequences from different geographic locations in the HIV Sequence Database (Table S1) and 58 sequences from our study on Hainan Island [30]. To improve the temporal signal of the likelihood of Markov chain Monte Carlo (MCMC) convergence of BEAST analysis, five sequences were eliminated based on R squared (R^2^) in TempEst v1.5.3 [31]. Finally, the Dataset-1 R^2^ value was 0.618. The maximum clade credibility (MCC) tree generated from Dataset-1 revealed that the Hainan strains formed a monophyletic clade, consisting of 53 Hainan sequences and one Beijing sequence. Dataset-2 was utilized (R^2^ = 0.310) to infer viral migration events among the age groups and cities on Hainan Island, which included the 53 Hainan sequences identified in the MCC analysis of Dataset-1.

We implanted Bayesian SkyGrid model in BEAST v1.10.5 to estimate the most recent common ancestor (tMRCA) and the evolutionary rate of CRF65_cpx [32]. The Hasegawa Kishino Yano plus Gamma plus Invariant Sites (HKY + G + I) nucleotide substitution model determined by Bayesian Information Criterion (BIC) using jModelTest v2.1.10 [33], along with a relaxed uncorrelated lognormal molecular clock model, was utilized for this analysis. The analysis for each dataset was 100 million generations in triplicate runs and sampling every 10,000 states. The triplicate BEAST runs were combined with LogCombiner v1.10.5pre, and the first 10% was discarded as burn-in. The estimated effective sample size (ESS) was over 200. The MCC trees were generated using Tree Annotator v1.10.5 and visualized in FigTree v1.4.3. A Bayesian stochastic search variable selection (BSSVS) procedure was used to calculate the Bayes factor (BF) to accurately describe the viral dissemination process [34]. This study only discussed the results with BF ≥ 3 and with posterior probability (*PP*) ≥ 0.80.

## Results

### The demographics of participants on hainan island

Table 1 provides an overview of the descriptive statistics from the study samples. The initial case of CRF65_cpx infection on Hainan Island was diagnosed in 2012, followed by 57 additional cases of the same genetic form in the subsequent nine years. The predominant proportion (89.66%) of these cases engaged in MSM, with the significant majority (96.55%) being male and demonstrating genotypic resistance (100%). The median age of the 58 participants was 25 years, ranging from 17 to 50 years. Of the 58 participants, 82.76% were single, 84.48% were from Haikou, 86.21% were recruited in 2021, 79.31% were on ART treatment, and 43.10% held educational qualifications of college graduate or above. The median of the baseline CD4 + T-cell counts was 269 cells/mm^3^, with an inter-quartile range (IQR) of 170 to 392.

**Table 1:** The characteristics of 58 patients infected with HIV-1 CRF65_cpx strain from Hainan, China

*Variable*	*Number (N)*	*Percent (%)*
Total	58	100.00
**Sex**		
Male	56	96.55
Female	2	3.45
**Age, years: median 25, rang (17,50)**		
17–24	26	44.83
25–40	25	43.10
Older than 41	7	12.07
**Sampling cities**		
Haikou	49	84.48
Sanya	5	8.62
Danzhou	2	3.45
Tunchang	1	1.72
Chengmai	1	1.72
**Sampling years**		
2018	2	3.45
2019	1	1.72
2020	4	6.90
2021	50	86.21
2022	1	1.72
**Risk factors**		
MSM	52	89.66
Heterosexual	3	5.17
Unknown	3	5.17
**Year of diagnosis**		
2012–2015	10	17.24
2016–2017	15	25.86
2018–2019	16	27.59
2020–2021	17	29.31
**Antiretroviral therapy**		
No	12	20.69
Yes	46	79.31
**Marital Status**		
Married and cohabiting	7	12.07
Single	48	82.76
Unknown	3	5.17
**Education**		
Junior high school or lower	11	18.97
High school	16	27.59
College graduate or above	25	43.10
Unknown	9	15.52
**Genotypic resistance**		
No	2	3.45
Yes	56	96.55
**V179D mutation**		
No	0	0
Yes	58	100.00
**Occupation**		
Worker	9	15.52
Clerks	8	13.79
Students	6	10.34
Unemployment	7	12.07
Others	13	22.41
Unknown	15	25.86
**Baseline CD4 + T- cell count, cells/mm**^**3**^**: median 269, IQR:170–392**		
< 200	10	17.20
200–350	18	31.00
301–500	16	27.60
> 500	5	8.60
Unknown	9	15.50

*Abbreviation*: *IQR* inter-quartile range, *MSM*, men who have sex with men

### The expansion of HIV-1 CRF65_cpx strain on hainan island

Of the 58 sequences, 39 (67.24%, 39/58) fell into the molecular network, forming a single cluster at a maximum pairwise genetic distance of 0.5%. Within this network, the nodes exhibited a median degree of 13, ranging from 1 to 25. Notably, 66.67% (26/39) of these nodes displayed more than four links, with all nodes exhibiting genotypic resistance. Within the established cluster, the median number of links was higher for individuals aged 25 to 41 years (*n* = 14) than for those aged 17 to 24 years (*n* = 8) and individuals above 40 years of age (*n* = 4).

**Fig. 1:**
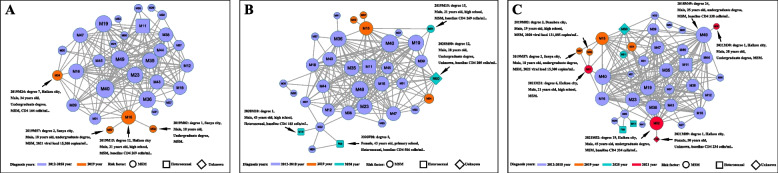
The dynamic transmission network of HIV-1 CRF65_cpx from 2019 to 2021 in Hainan, China. The edges (lines connecting the nodes) represent genetic relatedness. The nodes indicate HIV-1 patients or sequences. The characters in the node indicate gender: M denotes male, and F denotes female, while the number in the node represents the patient code. The colors indicate different diagnosis years: purple, 2012–2018; yellow, 2019; green, 2019; and red, 2021. The shapes indicate different transmission routes in (**A**), (**B**), and (**C**). The sizes of the geometrical figures represent the degree (lines connecting the nodes)

From 2019 to 2021, four individuals were added to the cluster annually, with those added in 2019 originating from three cities on Hainan Island. Of these four individuals, all of them were MSM aged 18–24 with a high school education or above, and three of them were directly genetic link to the same HIV infected people (M15) (Fig. 1A). The M15 exhibited a low CD4 + T-cell count of 269 cells/mm^3^ in December 2019, followed by a high viral load of 59,572 copies/mm^3^ in January 2020. In-depth tracking and investigation revealed that M15 engaged in multiple instances of both commercial and casual sex spanning nearly three months prior to diagnosis in 2019. Clinical surveillance subsequently revealed that medical treatment in this patient was ineffective in 2020. In the subsequent 2020 and 2021 networks, M15 connected directly with half of the new cases: M51 and M50 in 2020, and M31 and M52 in 2021 (Figs. 1B and C). Noticeably, the M15 most likely represented the key node driving the local CRF65_cpx transmission.

In 2020, four new nodes, one corresponding to a female individual and two to heterosexually infected individuals, were scattered throughout the network (Fig. 1B). In the 2020 network, a significant detail was that one new node (M31) connected with three others (M57, M15, and M02), which experienced a high viral load (Fig. 1C) and contributed to the HIV-1 CRF65_cpx epidemic complexity.

### The origin of HIV-1 CRF65_cpx in China and Hainan island

**Fig. 2:**
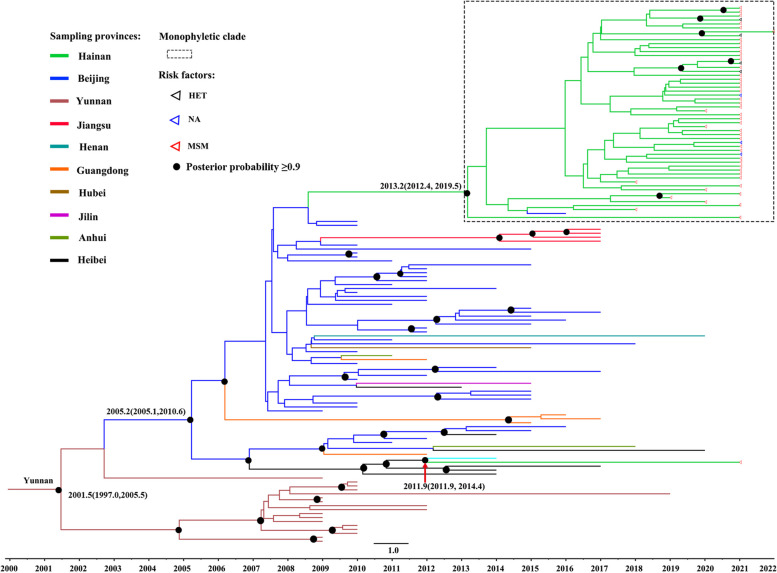
The Bayesian maximum clade credibility (MCC) tree of HIV-1 CRF65_cpx sequences. The MCC tree was constructed using Dataset-1, consisting of 136 sequences, of which 54 were from Hainan Province and 82 were downloaded from the HIV Sequence Database (Table S1). The colors of the rectangles indicate different provinces. The dotted rectangle represents the monophyletic clade, consisting of 53 Hainan sequences (Dataset-2) and one Beijing sequence. The colors of the triangles in the tip represent the risk factors. The values next to the black dots indicate the time of the most recent common ancestor (95%HPD intervals). Scale years are presented at the bottom of the figure. Note. HETs: Heterosexuals. MSM: Men who have sex with men

**Fig. 3:**
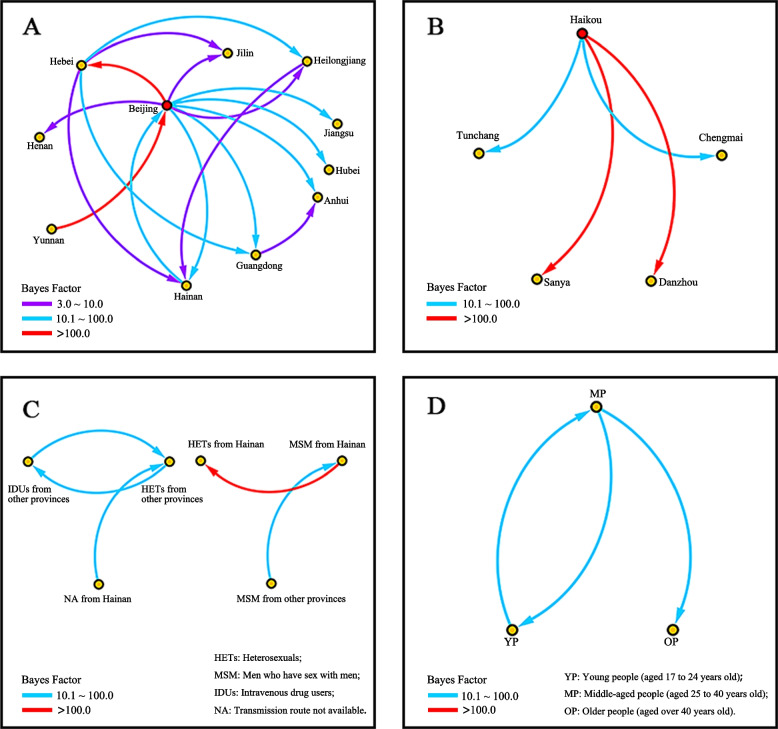
The inferred well-supported virus migration events of CRF65_cpx between the subgroups. Only results with Bayes factor (BF) ≥ 3 are presented. Arrows indicate the direction of the HIV-1 migration events. The colors were chosen to visually distinguish the different BF level values. **A** and **D** containing 136 sequences (Dataset-1) from different provinces, demonstrate viral migration events between provinces in China (3A) and the transmission pattern of HIV-1 CRF65_cpx (3D). **B** and **C** including 53 sequences only from Hainan (Dataset-2), present migration events among the age groups and cities in Hainan Province. Note. YP: Young people (aged 17 to 24 years old), MP: Middle-aged people (aged 25 to 40 years old), OP: Older people (OP, aged over 40 years old). MSM: Men who have sex with men. HETs: Heterosexuals. IDUs: Intravenous drug users. NA: Transmission route not available

The BEAST analysis revealed that the mean evolutionary rate of the CRF65_cpx strain was 2.115 × 10^–3^ subs/site/year [95%HPD: (1.691–2.591) × 10^–3^]. The MCC tree (Fig. 2) visually illustrated that CRF65_cpx originated in Yunnan Province with an inferred tMRCA year of 2001.5 (95% highest posterior density intervals (95%HPD): 1997.0–2005.5). Twelve of the eighteen Yunnan sequences found in the MCC tree were from HETs, and the remaining four came from non-MSM persons. The Beijing sequences were located between those of Yunnan and Hainan, of which eight (18.2%, 8/44) sequences were from MSM populations and 36 (81.8%, 36/44) sequences were from unknown populations. The estimated tMRCA of the Beijing clade was 2005.2 (95%HPD: 2005.1–2010.6). The investigation determined that the CRF65_cpx strain was most likely transmitted from Beijing to Hainan Island (BF = 17.4) around 2013.2 (95%HPD: 2012.4–2019.5). Additionally, the BFs of the viral migration from Hebei to Hainan (BF = 5.7) and from Heilongjiang to Hainan (BF = 4.7) were greater than 3 (Fig. 3A). Moreover, well-supported viral migrations from Beijing to Hebei (BF > 1000), Guangdong (BF = 96.6), Anhui (BF = 25.6), Jiangsu (BF = 16.8), Hubei (BF = 11.9), Henan (BF = 6.8), Jilin (BF = 6.5), and Heilongjiang (BF = 3.7) were observed in this study (Fig. 3A).

In the MCC tree (Fig. 2), the monophyletic lineage with a high PP (*PP* = 1.00) at the top consisted of 54 CRF65_cpx sequences, including 53 sequences from Hainan and one sequence from Beijing. The tMRCA of this lineage was estimated as 2013.2. Further analysis indicated that Haikou might be the epicenter of the CRF65_cpx strain on Hainan Island, which subsequently spread to Sanya (origin 2015.7, BF > 1000), Danzhou (2013.3, BF = 299.3), Chengmai (2014.5, BF = 27.0), and Tunchang (2013.6, BF = 16.3) (Fig. 3B and Fig. S2).

### The transmission dynamics between the age and risk subgroups

To reveal the historical migration patterns of CRF65_cpx strain in the age and risk subgroups, Bayesian phylogenetic analysis was conducted using Dataset-1 and Dataset-2, respectively. For the age subgroups, the results showed that the viral migration directions moved from middle-aged people (MP, aged 25 to 40 years) towards the older people (OP, aged over 40 years, BF = 17.6) and young people (YP, aged 17 to 24 years, BF = 14.6) groups (Fig. 3C). Additionally, well-supported transmission between the risk subgroups was inferred from MSM in other provinces to Hainan MSM (BF = 29.1) (Fig. 3D). Migration events with strong BF support (BF > 1000) were observed on Hainan Island, specifically from MSM to HETs (Fig. 3D).

## Discussion

This study conducted a comprehensive analysis to elucidate the origin and dissemination patterns of HIV-1 CRF65_cpx on Hainan Island, China. The introduction of the CRF65_cpx strain occurred via the MSM population in Beijing around February 2013. Subsequently, the infection propagated within the local MSM, leading to the emergence of a substantial and stable transmission cluster. Notably, Haikou city became the hub for the rapid spread of CRF65_cpx on Hainan Island. The transmission dynamics indicated a pattern from MSM to HETs. Particularly, MSM aged 25 to 40 years played a crucial role in bridging the transmission gap between younger and older MSM.

This study indicated that the majority of individuals infected with the CRF65_cpx strain were MSM, with 43.10% possessing a college degree or higher. One plausible explanation was the fact that MSM were the predominant population affected by HIV on Hainan Island. Furthermore, individuals identifying as MSM represented a substantial 72.72% of those afflicted with HIV who possessed a college education or higher [35]. Consequently, targeted interventions for higher education MSM should be prioritized to effectively reduce HIV transmission. Additionally, the network analysis showed that over two-thirds of HIV-positive individuals were organized into only one cluster, including 39 members, while 66.7% of the nodes had more than four links. A higher clustering rate was observed for CRF65_cpx with a lower genetic cutoff than CRF01_AE, CRF07_BC, and CRF08_BC in other regions of China [36, 37]. This finding demonstrates the close interconnection within the population affected by this viral strain. It suggests that targeted interventions can be employed for individuals infected with CRF65_cpx. Increasing testing efforts among individuals with high-risk contact with those infected by CRF65_cpx, proactively identifying infected cases and early treatment are important strategies to mitigate secondary transmission. Furthermore, continued monitoring is essential for CRF65_cpx cluster expansion.

From 2019 to 2021, the cluster exhibited a consistent expansion pattern. M15 may hold a pivotal position as a central node or potentially act as a super-spreader in the transmission dynamics of CRF65_cpx on Hainan Island. The Chinese CDC's guidelines for HIV Transmission Network recommend that large clusters (≥ 10 nodes) and nodes displaying high viral loads or drug resistance should be considered as key clusters and nodes for surveillance and intervention [38]. Therefore, it is essential to identify key clusters and individuals among HIV-positive patients. The results of this study showed consistent network expansion. Additionally, due to commercial or casual sex and a high viral load, the M15 patient possibly played a key role in local CRF65_cpx transmission. The previous research demonstrated that nodes with unusual characteristics likely represented opportunities for breaking important transmission chains [39]. Our findings suggest that the patients in the large cluster, especially the M15 case, should be the primary intervention target. For example, drug adherence monitoring and follow-up should be enhanced for key populations, and viral load assessments should be performed for those living with HIV.

Our findings highlight the therapeutic difficulties faced by patients infected with CRF65_cpx strains. All the CRF65_cpx sequences on Hainan Island carried the V179D mutation, which was in line with findings from an earlier CRF65_cpx investigation [15]. However, the prevalence of this mutation was significantly higher in CRF65_cpx than in other subtypes, such as CRF01_AE (7.1%) [40], and subtype F (4.1%) [14]. The V17D mutation is a polymorphic accessory mutation, leading to a twofold reduction in NVP, EFV, ETR, and RPV susceptibility [41]. Additionally, the V179D and K103R mutation co-occurrence caused a 15-fold decrease in NVP and EFV susceptibility [18]. The observed synergistic effect of the V179D and V106I combination indicated a reduction in NVP and EFV susceptibility [42]. Based on the findings, implementing intervention measures is recommended for all HIV-positive patients harboring V179D mutations.

Similar to previous analyses, this study confirmed that CRF65_cpx originated from individuals who acquired HIV via heterosexual transmission in Yunnan Province around 2001.5 [16]. Approximately four years later, the CRF65_cpx strain disseminated to the MSM population in Beijing. This study established Beijing as a significant epicenter in the spread of the CRF65_cpx strain to other regions, as evidenced by a high BF. It was hypothesized that the CRF65_cpx strain most likely arrived in Hainan via MSM in Beijing in September 2013. Beijing, the nation's capital, is home to a sizable MSM population, which accounted for 73.9% of newly diagnosed HIV/AIDS infections in 2016 [43]. The high mobility of the MSM population has contributed to the rapid dissemination of diverse HIV-1 strains throughout China. The phenomenon of novel strains circulating within the MSM population and subsequently leading to cross-regional transmission, such as CRF01_AE [44], CRF07_BC [45], and CRF55_01B [46], is evident. In recent years, MSM has been a major factor in the disease prevalence in Hainan [35]. As a tropical tourist destination, Hainan Island attracts individuals from across the country, including MSM. Consequently, they have played a role in the emergence of new HIV CRFs and URFs, heightening the intricacy of the HIV epidemic among MSM populations in Hainan Island. It is worth noting that, compared to HETs, the CRF65_cpx strains in MSM population lack the protective epitopes of HLA-B*2702 and HLA-B*5103. This observation implies the potential adaptability and accelerated disease progression of this strain in MSM population [16]. Therefore, a comprehensive investigation into the disease progression and drug resistance aspects of the CRF65_cpx strain are warranted.

On Hainan Island, Haikou represents the epicenter of the spread of the CRF65_cpx strains to other cities via homosexual contact in different years, including Sanya, Danzhou and Chengmai. Given that Haikou serves as the provincial capital of Hainan Province and harbors the largest number of HIV-1 cases, especially among the MSM, its role as a hub is paramount. Sanya follows closely as another significant city in this context. This pattern could likely be attributed to the spillover effect from Haikou to neighboring region on Hainan Island. Additionally, CRF65_cpx has been detected across diverse age groups and within heterosexual populations, underscoring the complexity of the CRF65_cpx epidemic. Consequently, it is evident that the landscape of the CRF65_cpx strains is multifaceted. Therefore, maintaining a robust molecular surveillance system for CRF65_cpx strains, with a particular emphasis on the MSM population, is necessary.

The CRF65_cpx epidemic in Hainan is highly complex. This study revealed the presence of CRF65_cpx strains in both HETs and a broad MSM age group. The Bayesian analysis inferred that viral migration predominantly occurred from MP towards EP and YP, suggesting that MP may potentially play a pivotal role in CRF65_cpx dissemination. Among young MSM, factors such as limited HIV awareness, underestimation of personal risk, peer influence, interaction with older MSM, sexual preferences, and engagement in transactional sex have been identified as drivers of HIV-1 transmission [47, 48]. Young MSM might face additional vulnerabilities, such as sexual coercion, stigma, and social exclusion, further underscoring the significant influence exerted by older MSM on their younger counterparts.

The study presented several potential limitations. First, sampling bias was introduced because the inclusion criteria relied on patients attending the sampling hospital for the first time or keeping appointments for follow-ups within the recruitment period. Second, the relative sample size was a constraint due to the emerging nature of CRF65_cpx as an HIV-1 strain and the limited sequence availability. Third, there was temporal bias during the sampling period, since a significant proportion of the Hainan sequences were collected in 2021. However, we included all CRF65_cpx sequences downloaded from the HIV Sequence Database to minimize sampling year bias.

In conclusion, this study provides a thorough comprehension of the origin and transmission dynamics of CRF65_cpx on Hainan Island. The results indicate that the CRF65_cpx strain was introduced to Hainan Island by MSM from Beijing around 2013, followed by local dissemination. Notably, MSM aged 25 to 40 years play a pivotal role in bridging the gap between the younger and older MSM population. Given the implications for public health, it is essential to give immediately prioritize to molecular surveillance of the CRF65_cpx strain. This will make it easier to create sensible guidelines and recommendations for the early intervention and containment of the CRF65_cpx pandemic, especially for a population as migratory as the MSM on Hainan Island.

### Supplementary Information

**Additional file 1: Table S1.** List of 83 sequences of CRF65_cpx downloaded from the HIV Sequence Database. **Figure S1.** Maximum-likelihood (ML) phylogenetic tree of HIV-1 *pol* sequences. The ML tree, containing 711 sequences, was constructed with all available subtype C and CRF65_cpx sequences from China, and references of other subtypes (A1, A2, B, B', D, K, F1, F2, H, N, CRF01_AE, CRF07_BC, CRF08_BC and other CRFs) from the HIV Sequence Database by FastTree v2.2.10. Group N was set as outgroup. Background colours represent subtypes: the green denotes reference sequences (except subtype C and CRF65_cpx), the blue represents subtype C and the yellow indicates CRF65_cpx. Under the yellow background, the blue clade represents CRF65_cpx from Hainan Island, the yellow clade represents CRF65_cpx from other provinces, the red clade represents the sequences used for CRF65_cpx identification [1], and the dark brown clade denotes the CRF65_cpx misclassified as subtype C. The numbers near the red dots represent the Shimodaira-Hasegawa (SH)-like node support values. The tip label consists of subtype, sampling year, and GenBank accession. **Figure S2.** Bayesian maximum clade credibility (MCC) tree of Hainan monophyletic clade. The MCC tree was constructed using Dataset-2, including 55 sequences from Hainan Island. The values next to the green dots indicate the times of the most recent common ancestors. Line colors indicate different cities within Hainan Island. Scale years are shown at the bottom of the figure.

## Data Availability

This study analyzed 58 HIV-1 CRF65_cpx sequences on Hainan Island, which were submitted to the HIV Sequence Database (http://www.hiv.lanl.gov/). The GenBank accessions of these sequences are OP830984, OP831061, OP831108, OP830987, OP831006, OP831102, OP831132 and OR606459-OR606509. The publicly available sequences used in this current study were downloaded from the HIV Sequence Database, the GenBank accessions of which are shown in Table S1.
