# Supplementary material for: The origin, dissemination, and molecular networks of HIV-1 CRF65_cpx strain in Hainan Island, China
Source: BMC Infect Dis. 2024 Mar 1;24:269. doi: 10.1186/s12879-024-09101-w (PMC10905908; doi:10.1186/s12879-024-09101-w)
Supplement: Supplementary file 1 — Additional file 1: Table S1. List of 83 sequences of CRF65_cpx downloaded from the HIV Sequence Database. Figure S1. Maximum-likelihood (ML) phylogenetic tree of HIV-1 pol sequences. The ML tree, containing 711 sequences, was constructed with all available subtype C and CRF65_cpx sequences from China, and references of other subtypes (A1, A2, B, B', D, K, F1, F2, H, N, CRF01_AE, CRF07_BC, CRF08_BC and other CRFs) from the HIV Sequence Database by FastTree v2.2.10. Group N was set as outgroup. Background colours represent subtypes: the green denotes reference sequences (except subtype C and CRF65_cpx), the blue represents subtype C and the yellow indicates CRF65_cpx. Under the yellow background, the blue clade represents CRF65_cpx from Hainan Island, the yellow clade represents CRF65_cpx from other provinces, the red clade represents the sequences used for CRF65_cpx identification [1], and the dark brown clade denotes the CRF65_cpx misclassified as subtype C. The numbers near the red dots represent the Shimodaira-Hasegawa (SH)-like node support values. The tip label consists of subtype, sampling year, and GenBank accession. Figure S2. Bayesian maximum clade credibility (MCC) tree of Hainan monophyletic clade. The MCC tree was constructed using Dataset-2, including 55 sequences from Hainan Island. The values next to the green dots indicate the times of the most recent common ancestors. Line colors indicate different cities within Hainan Island. Scale years are shown at the bottom of the figure. [file 12879_2024_9101_MOESM1_ESM.docx]

**Table S1. List of 83 sequences of CRF65_cpx downloaded from the HIV Sequence Database**

| **Accession number** | **Subtype in the database** | **Sampling year** | **Subtype reclassified** | **Sex** | **Risk factor** | **Sampling province** | **In the Bayesian dataset-1** |
| --- | --- | --- | --- | --- | --- | --- | --- |
| JQ658554 | C | 2009 | CRF65_cpx | NA | non-MSM | Yunnan | Yes |
| JQ658555 | CRF65_cpx | 2009 | CRF65_cpx | M | HET | Yunnan | Yes |
| JQ658570 | C | 2009 | CRF65_cpx | NA | non-MSM | Yunnan | Yes |
| JQ658585 | C | 2009 | CRF65_cpx | NA | non-MSM | Yunnan | Yes |
| JQ658731 | C | 2010 | CRF65_cpx | NA | non-MSM | Yunnan | Yes |
| JQ658756 | C | 2010 | CRF65_cpx | F | HET | Yunnan | Yes |
| JQ898223 | CRF65_cpx | 2009 | CRF65_cpx | M | IDU | Yunnan | Yes |
| KC183778 | CRF65_cpx | 2011 | CRF65_cpx | M | MSM | Anhui | Yes |
| KC870027 | CRF65_cpx | 2010 | CRF65_cpx | F | HET | Yunnan | Yes |
| KC870028 | CRF65_cpx | 2010 | CRF65_cpx | F | HET | Yunnan | Yes |
| KF714310 | C | 2009 | CRF65_cpx | F | HET | Yunnan | Yes |
| KF714326 | C | 2009 | CRF65_cpx | M | HET | Yunnan | Yes |
| KF714341 | C | 2009 | CRF65_cpx | F | HET | Yunnan | Yes |
| KF714363 | C | 2010 | CRF65_cpx | F | HET | Yunnan | Yes |
| KF714374 | C | 2010 | CRF65_cpx | F | HET | Yunnan | Yes |
| KF714427 | C | 2011 | CRF65_cpx | M | HET | Yunnan | Yes |
| KF714461 | C | 2012 | CRF65_cpx | F | HET | Yunnan | Yes |
| KF714479 | C | 2012 | CRF65_cpx | F | HET | Yunnan | Yes |
| KP250691 | C | 2009 | CRF65_cpx | M | MSM | Beijing | Yes |
| KP250714 | C | 2010 | CRF65_cpx | M | MSM | Beijing | Yes |
| KP698503 | CRF65_cpx | 2010 | CRF65_cpx | M | MSM | Beijing | Yes |
| KP698504 | CRF65_cpx | 2010 | CRF65_cpx | M | MSM | Beijing | Yes |
| KP698505 | CRF65_cpx | 2010 | CRF65_cpx | M | MSM | Beijing | Yes |
| KP698506 | CRF65_cpx | 2011 | CRF65_cpx | M | NA | Beijing | Yes |
| KP698507 | CRF65_cpx | 2012 | CRF65_cpx | M | MSM | Beijing | Yes |
| KP698508 | CRF65_cpx | 2012 | CRF65_cpx | M | MSM | Beijing | Yes |
| KT379558 | C | 2012 | CRF65_cpx | M | MSM | Guangdong | Yes |
| KX198586 | CRF65_cpx | 2013 | CRF65_cpx | M | MSM | Hebei | Yes |
| KY713419 | CRF65_cpx | 2016 | CRF65_cpx | M | MSM | Beijing | Yes |
| MF941253 | C | 2014 | CRF65_cpx | NA | NA | Heilongjiang | Yes |
| MG706490 | CRF65_cpx | 2014 | CRF65_cpx | M | NA | Hebei | Yes |
| MG706508 | CRF65_cpx | 2014 | CRF65_cpx | M | NA | Hebei | Yes |
| MG706585 | CRF65_cpx | 2014 | CRF65_cpx | M | NA | Hebei | Yes |
| MH051841 | CRF65_cpx | 2015 | CRF65_cpx | M | MSM | Jilin | Yes |
| MH921146 | CRF65_cpx | 2016 | CRF65_cpx | NA | NA | Beijing | Yes |
| MH921170 | CRF65_cpx | 2016 | CRF65_cpx | NA | NA | Beijing | Yes |
| MK771306 | CRF65_cpx | 2018 | CRF65_cpx | NA | NA | Beijing | Yes |
| MN633695 | C | 2018 | CRF65_cpx | M | MSM | Anhui | Yes |
| MN796685 | C | 2010 | CRF65_cpx | NA | NA | Beijing | Yes |
| MN796845 | C | 2010 | CRF65_cpx | NA | NA | Beijing | Yes |
| MN796862 | C | 2010 | CRF65_cpx | NA | NA | Beijing | Yes |
| MN797005 | C | 2010 | CRF65_cpx | NA | NA | Beijing | Yes |
| MN797033 | C | 2010 | CRF65_cpx | NA | NA | Beijing | Yes |
| MN797080 | C | 2010 | CRF65_cpx | NA | NA | Beijing | Yes |
| MN797287 | C | 2010 | CRF65_cpx | NA | NA | Beijing | Yes |
| MN797602 | C | 2011 | CRF65_cpx | NA | NA | Beijing | Yes |
| MN797768 | C | 2011 | CRF65_cpx | NA | NA | Beijing | Yes |
| MN797881 | C | 2011 | CRF65_cpx | NA | NA | Beijing | Yes |
| MN797978 | C | 2011 | CRF65_cpx | NA | NA | Beijing | Yes |
| MN798159 | C | 2012 | CRF65_cpx | NA | NA | Beijing | Yes |
| MN798282 | C | 2012 | CRF65_cpx | NA | NA | Beijing | Yes |
| MN798328 | C | 2012 | CRF65_cpx | NA | NA | Beijing | Yes |
| MN798343 | C | 2012 | CRF65_cpx | NA | NA | Beijing | Yes |
| MN798538 | C | 2012 | CRF65_cpx | NA | NA | Beijing | Yes |
| MN798567 | C | 2012 | CRF65_cpx | NA | NA | Beijing | Yes |
| MN798697 | C | 2012 | CRF65_cpx | NA | NA | Beijing | Yes |
| MN798723 | C | 2012 | CRF65_cpx | NA | NA | Beijing | Yes |
| MN799854 | C | 2014 | CRF65_cpx | NA | NA | Beijing | Yes |
| MN800203 | C | 2014 | CRF65_cpx | NA | NA | Beijing | Yes |
| MN800815 | C | 2015 | CRF65_cpx | NA | NA | Beijing | Yes |
| MN801034 | C | 2015 | CRF65_cpx | NA | NA | Beijing | Yes |
| MN801152 | C | 2015 | CRF65_cpx | NA | NA | Beijing | Yes |
| MN801272 | C | 2015 | CRF65_cpx | NA | NA | Beijing | Yes |
| MN801283 | C | 2015 | CRF65_cpx | NA | NA | Beijing | Yes |
| MN801293 | C | 2015 | CRF65_cpx | NA | NA | Beijing | Yes |
| MN801367 | C | 2015 | CRF65_cpx | NA | NA | Beijing | Yes |
| MN802019 | C | 2015 | CRF65_cpx | NA | NA | Beijing | Yes |
| MN802154 | C | 2015 | CRF65_cpx | NA | NA | Beijing | Yes |
| MN802733 | C | 2017 | CRF65_cpx | NA | NA | Beijing | Yes |
| MN802893 | C | 2017 | CRF65_cpx | NA | NA | Beijing | Yes |
| MT986497 | CRF65_cpx | 2017 | CRF65_cpx | M | MSM | Jiangsu | Yes |
| MT986550 | CRF65_cpx | 2017 | CRF65_cpx | M | MSM | Jiangsu | Yes |
| MT986555 | CRF65_cpx | 2017 | CRF65_cpx | M | MSM | Jiangsu | Yes |
| MT986570 | CRF65_cpx | 2017 | CRF65_cpx | M | MSM | Jiangsu | Yes |
| MW948931 | CRF65_cpx | 2015 | CRF65_cpx | M | NA | Guangdong | Yes |
| MW949972 | CRF65_cpx | 2016 | CRF65_cpx | M | NA | Guangdong | Yes |
| MW951506 | CRF65_cpx | 2017 | CRF65_cpx | M | NA | Guangdong | Yes |
| MW952269 | CRF65_cpx | 2017 | CRF65_cpx | M | NA | Guangdong | Yes |
| MW955500 | CRF65_cpx | 2012 | CRF65_cpx | M | NA | Guangdong | Yes |
| MZ956105 | C | 2020 | CRF65_cpx | M | MSM | Hebei | Yes |
| OL684441 | C | 2015 | CRF65_cpx | NA | NA | Hubei | No |
| OM454528 | CRF65_cpx | 2019 | CRF65_cpx | NA | NA | Yunnan | Yes |
| OP191603 | C | 2020 | CRF65_cpx | NA | NA | Henan | No |

Abbreviations: M: male; F: female; HET: heterosexual; IDU: intravenous drug user; MSM: men who have sex with men; NA: not available. In the table, 53 CRF65_cpx sequences were misclassified as subtype C.


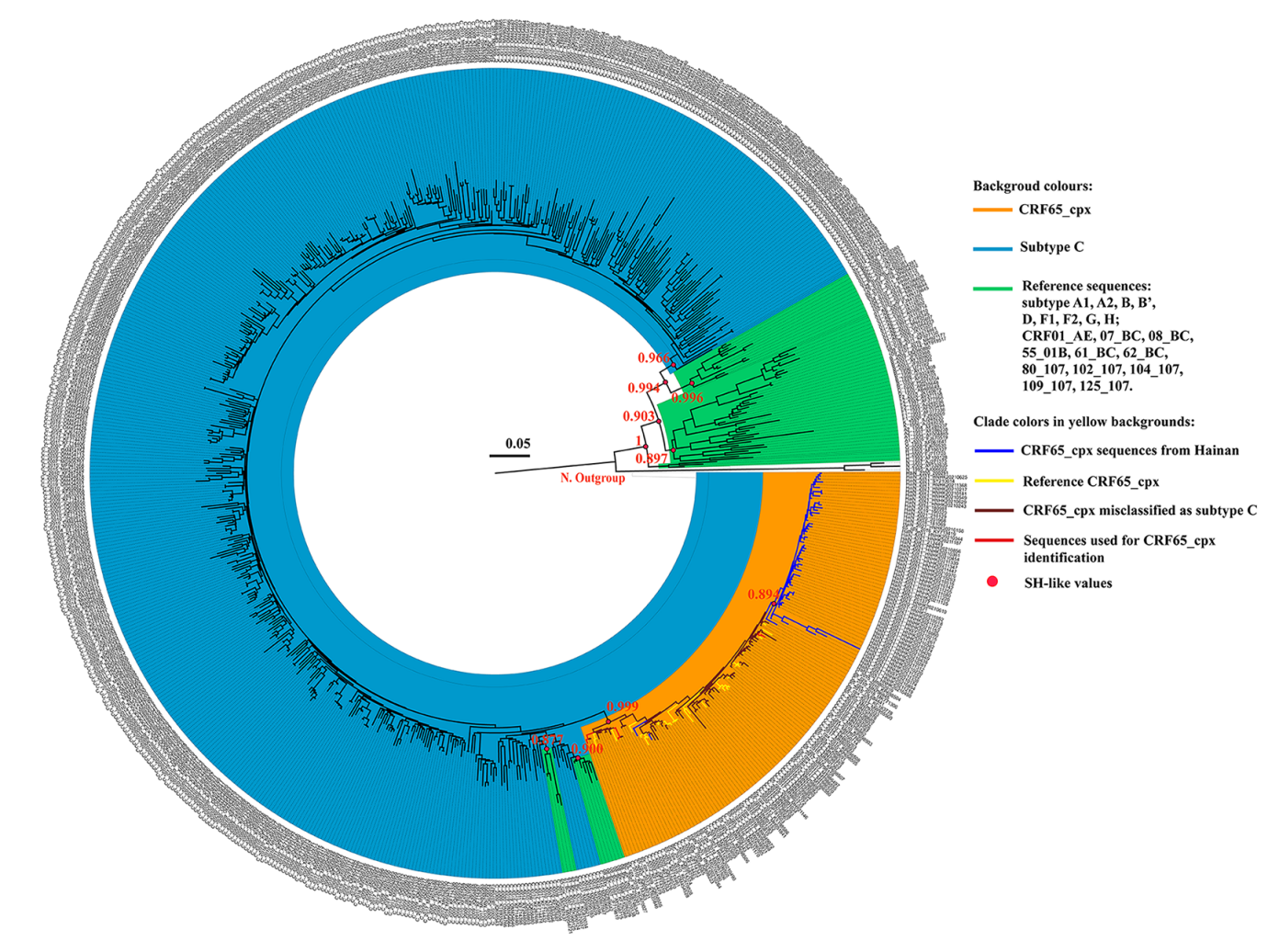


**Figure S1**. Maximum-likelihood (ML) phylogenetic tree of HIV-1 *pol* sequences. The ML tree, containing 711 sequences, was constructed with all available subtype C and CRF65_cpx sequences from China, and references of other subtypes (A1, A2, B, B', D, K, F1, F2, H, N, CRF01_AE, CRF07_BC, CRF08_BC and other CRFs) from the HIV Sequence Database by FastTree v2.2.10. Group N was set as outgroup. Background colours represent subtypes: the green denotes reference sequences (except subtype C and CRF65_cpx), the blue represents subtype C and the yellow indicates CRF65_cpx. Under the yellow background, the blue clade represents CRF65_cpx from Hainan Island, the yellow clade represents CRF65_cpx from other provinces, the red clade represents the sequences used for CRF65_cpx identification [1], and the dark brown clade denotes the CRF65_cpx misclassified as subtype C. The numbers near the red dots represent the Shimodaira-Hasegawa (SH)-like node support values. The tip label consists of subtype, sampling year, and GenBank accession.

**References**

1. Feng Y, Wei H, Hsi J, Xing H, He X, Liao L, et al. Identification of a novel HIV Type 1 circulating recombinant form (CRF65_cpx) composed of CRF01_AE and subtypes B and C in Western Yunnan, China. AIDS Res Hum Retroviruses. 2014 Jun;30(6):598-602.


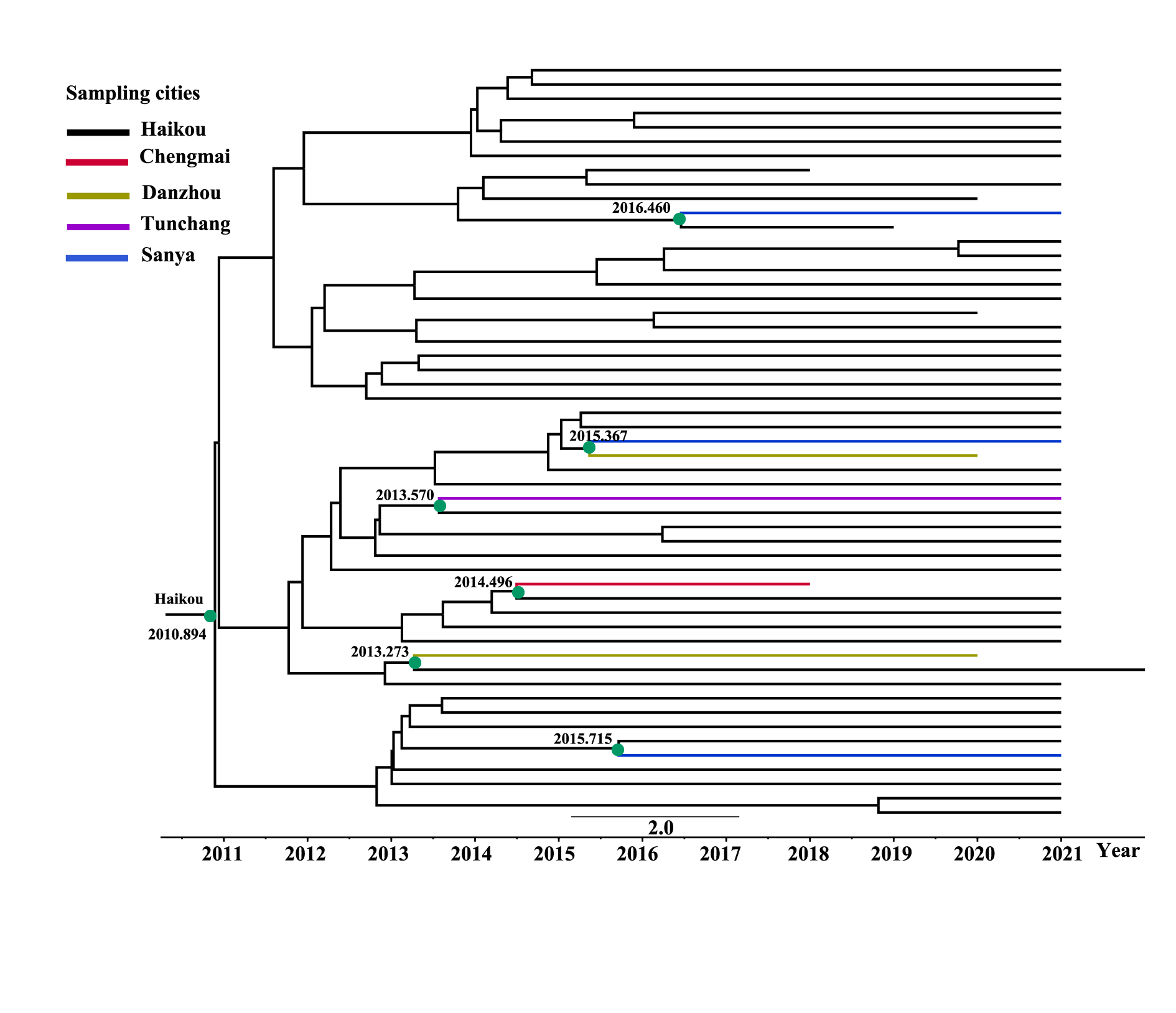


**Figure S2.** Bayesian maximum clade credibility (MCC) tree of Hainan monophyletic clade. The MCC tree was constructed using Dataset-2, including 55 sequences from Hainan Island. The values next to the green dots indicate the times of the most recent common ancestors. Line colors indicate different cities within Hainan Island. Scale years are shown at the bottom of the figure.
